# Azelnidipine, Not Amlodipine, Induces Secretion of Vascular Endothelial Growth Factor From Smooth Muscle Cells and Promotes Endothelial Tube Formation

**DOI:** 10.14740/cr352w

**Published:** 2014-10-06

**Authors:** Akira Kawamura, Shin-ichiro Miura, Yoshino Matsuo, Hiroyuki Tanigawa, Keijiro Saku

**Affiliations:** a Department of Cardiology, Fukuoka University School of Medicine, Fukuoka 814-0180, Japan; b Department of Molecular Cardiovascular Therapeutics, Fukuoka University School of Medicine, Fukuoka 814-0180, Japan

**Keywords:** Calcium channel blocker, Vascular endothelial growth factor, Smooth muscle cell, Endothelial cell tube formation, Nuclear factor-kappa B

## Abstract

**Background:**

We previously reported that the calcium channel blocker (CCB) nifedipine-induced secretion of vascular endothelial growth factor (VEGF) from human coronary smooth muscle cells (HCSMCs) promoted human coronary endothelial cell (HCEC) tube formation. Therefore, we analyzed whether other CCBs, azelnidipine and amlodipine, also induced the secretion of VEGF and promoted HCEC tube formation, and the underlying molecular mechanisms.

**Methods:**

To evaluate the tube formation, HCECs were grown on Matrigel for 18 hours in the supernatants from HCSMCs that had been treated with different kinds of reagents. Concentrations of VEGF in cultured HCSMCs were determined by specific enzyme immunoassays. Nuclear extracts from HCSMCs were prepared, and nuclear factor-kappa B (NF-κB) activation was measured by EZ-Detect^TM^ Transcription Factor Kits for NF-κB p50 or p65.

**Results:**

Although azelnidipine dose-dependently stimulated the significant secretion of VEGF from HCSMCs and this stimulation was abolished by a protein kinase C inhibitor, amlodipine-induced secretion of VEGF was significantly lower than that induced by azelnidipine. The medium derived from azelnidipine (at up to 2 μM)-treated HCSMCs led to HCEC tube formation, whereas that obtained with amlodipine did not. Azelnidipine-induced tube formation was blocked by an inhibitor of kinase insert domain-containing receptor/fetal liver kinase-1 tyrosine kinase. Azelnidipine at up to 2 μM induced NF-κB activation.

**Conclusions:**

Azelnidipine, but not amlodipine, stimulated the secretion of VEGF from HCSMCs and induced HCEC tube formation. This secretion is mediated at least in part via the activation of NF-κB. Azelnidipine may have a novel beneficial effect in improving coronary microvascular blood flow in addition to its main effect of lowering blood pressure.

## Introduction

Calcium channel blockers (CCBs) are widely used for the treatment of hypertension and coronary artery disease (CAD) because these drugs induce effective vasodilation, including in coronary arteries [1]. Most hypertensive patients require two or more antihypertensive agents to reach their target blood pressure (BP) [2]. A combination of angiotensin II type 1 receptor blockers (ARB)/CCB is currently the best therapy for preventing cardiovascular disease. Such combinations include many kinds of ARB and CCBs. Thus, differences in safety and efficacy among ARB/CCBs depend on the kind of ARB or CCB. Recent studies have shown that not all ARBs have the same effects, and some benefits conferred by ARBs may not be class (or common) effects, but rather molecular (or differential) effects [3]. Furthermore, CCBs also have molecular effects independent of BP-lowering. In fact, it has been reported that CCBs exert cardiovascular protective actions, including the reduction of oxidative stress and the proinflammatory response, the upregulation of endothelial superoxide dismutase expression, and the stimulation of nitric oxide (NO) [4-7].

Angiogenesis, the process of postnatal neovascularization, is a critical component of several human diseases, including CAD, cancer, diabetic microvascular disease and rheumatoid arthritis [8]. We previously reported that nifedipine-induced protein kinase C (PKC) activation occurs through bradykinin (BK) B2 receptor, which then increases the secretion of vascular endothelial growth factor (VEGF) from human coronary artery smooth muscle cells (HCSMCs) [9]. This VEGF secretion directly induced human coronary endothelial cell (HCEC) tube formation via a VEGF receptor kinase insert domain-containing receptor/fetal liver kinase-1 (KDR/Flk-1)/NO pathway. Therefore, we analyzed whether the CCBs azelnidipine and amlodipine have molecular effects with regard to angiogenesis.

## Methods

### Materials

The following reagents were purchased: a specific inhibitor of VEGF receptor, KDR/Flk-1 tyrosine kinase (4-[(4’-chloro-2’-fluoro)phenylaminol]-6,7-dimethoxy-quinazoline; Tki), an inhibitor of PKC, Go6980 (3-[1-(3-dimethylaminopropyl)-5-methoxyindol-3-yl]-3-(1H-indol-3-yl) maleimide) from Calbiochem, and BK from Peptide Institute Inc.

### Cell culture

HCSMCs and HCECs were purchased from Clonetics. HCSMCs and HCECs were cultured and used from three to six passages in media supplemented with 5% fetal bovine serum (FBS), penicillin/streptomycin, and smooth muscle cell-growth or endothelial cell-growth supplement (Takara Co., Osaka, Japan) at 37 °C under 5% CO_2_. HCSMCs and HCECs without cell-growth supplement were used in the experiments.

### Cell proliferation assay

HCSMCs and HCECs were plated on a 96-well plate and cultured under 5% serum conditions. After 48 h, the cells were cultured for 18 h in the presence or absence of different kinds of CCBs in medium supplemented with 0.2% FBS and without cell-growth supplement at 37 °C under 5% CO_2_. The cells were stained with CellTiter 96 One Solution Reagent (a novel tetrazolium compound (3-(4,5-dimethylthiazol-2-yl)-5-(3-carboxymethoxyphenyl)-2-(4-sulfophenyl)-2H-tetrazolium, inner salt; MTS assay) (Promega) for 4 h at 37 °C under 5% CO_2_, and absorbance at 490 nm was recorded with 96-well plate reader.

### Angiogenesis assay on Matrigel

An angiogenesis assay on Matrigel was performed as described previously [9, 10]. Briefly, the matrix gels (Chemicon International, Inc., Temecula, CA, USA) were allowed to polymerize in the plate. ECs were seeded and grown on Matrigel for 18 h in a humidified 37 °C under 5% CO_2_ incubator in the supernatants from SMCs that had been treated with different kinds of reagents in medium supplemented with 0.5% FBS and without growth supplement. After washing, tube formation was observed using a light microscope, and pictures were captured with a computer system. We performed a “pixel analysis” of the area of tube formation according to a procedure described previously [9, 10].

### Enzyme immunoassay

Concentrations of VEGF in cultured HCSMCs that had been treated with different kinds of reagents in medium supplemented with 0.5% FBS and without growth supplement for 18 h in a humidified at 37 °C were determined as described previously [11] in duplicate by specific enzyme immunoassays (R&D Systems) according to the manufacturer’s instructions.

### Measurement of nuclear factor-kappa B (NF-κB) activation

HCSMCs were grown under serum-free conditions for 24 h and stimulated with 50 ng/mL interleukin (IL)-1β with the indicated concentration of CCB. Nuclear extracts from HCSMCs were prepared and NF-κB activation was measured by EZ-Detect^TM^ Transcription Factor Kits for NF-κB p50 or p65 (Pierce).

### Statistical analysis

The results are expressed as the mean ± standard deviation of three or more independent determinations. Significant differences in measured values were evaluated with an analysis of variance using the paired or unpaired Student’s *t*-test, as appropriate. Statistical significance was set at P < 0.05. Data were analyzed using commercially available statistical software (Statview-J 5.0; Abacus Concepts Inc.).

## Results

### Cell proliferation in HCSMCs or HCECs using CCBs

**Figure 1:**
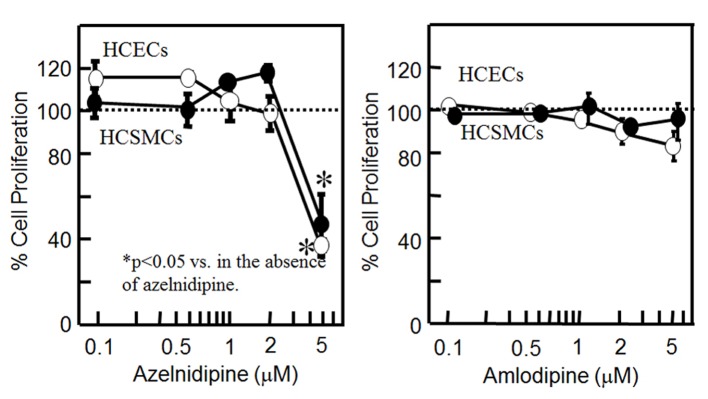
Cell proliferation in HCSMCs or HCECs with or without the indicated concentrations of azelnidipine (A) and amlodipine (B) for 18 h as assessed by an MTS assay. Open and closed circles indicate HCECs and HCSMCs, respectively. Graph shows the % cell proliferation compared with that in the untreated control sample (100%). *P < 0.05 vs. control.

To analyze whether CCB might stimulate or suppress cell proliferation, we analyzed whether CCB induced the proliferation of HCSMCs and HCECs (Fig. 1). Since the concentration of nifedipine used in our previous report was from 0.1 to 5 μM [9], we used the same concentration range for the CCBs. Although azelnidipine at up to 2 μM did not affect the proliferation of HCSMCs or HCECs, at 5 μM it significantly inhibited the proliferation of HCSMCs and HCECs. On the other hand, amlodipine at up to 5 μM did not affect the proliferation of either type of cell.

### VEGF secretion from HCSMCs using CCBs

**Figure 2:**
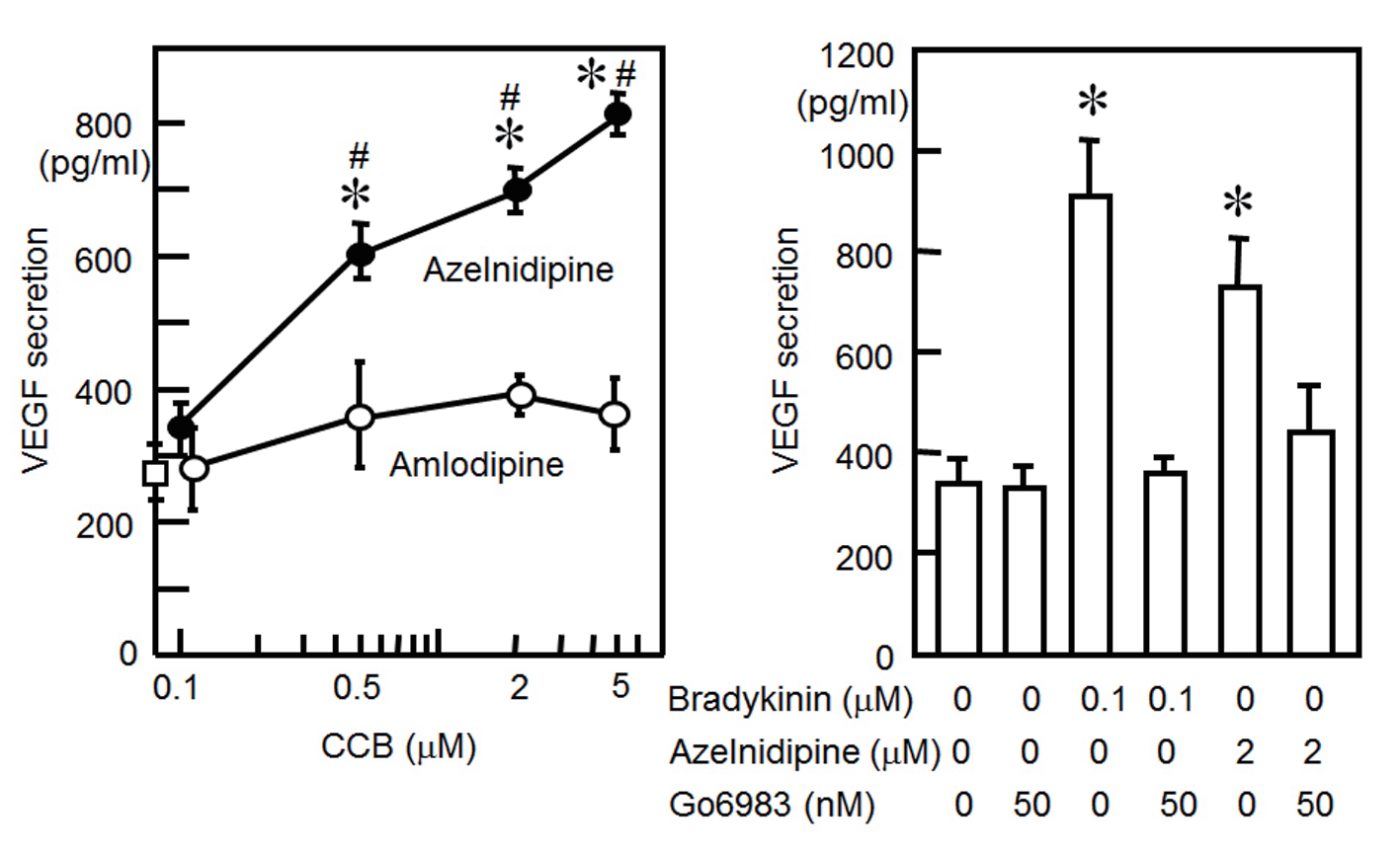
(A) VEGF secretion from HCSMCs with or without the indicated concentrations of azelnidipine (closed circle) and amlodipine (open circles). *P < 0.05 vs. no treatment. #P < 0.05 vs. amlodipine. (B) VEGF secretion from HCSMCs with or without bradykinin, azelnidipine or Go6983. *P < 0.05 vs. no treatment.

We examined whether CCBs increased the release of VEGF from HCSMCs. As shown in Fig. 2A, azelnidipine induced a significant increase in the release of VEGF from HCSMCs: concentration-dependent increases in VEGF secretion were observed, whereas amlodipine did not increase VEGF secretion.

Since stretching and bradykinin increased the secretion of VEGF through PKC activation [12, 13], we determined whether azelnidipine induces the secretion of VEGF through a PKC pathway (Fig. 2B). We preincubated cells for 1 h with the selective PKC inhibitor Go6983. This inhibitor blocked the azelnidipine (2 μM)-induced secretion of VEGF from HCSMCs.

### NF-κB activation using azelnidipine

**Figure 3:**
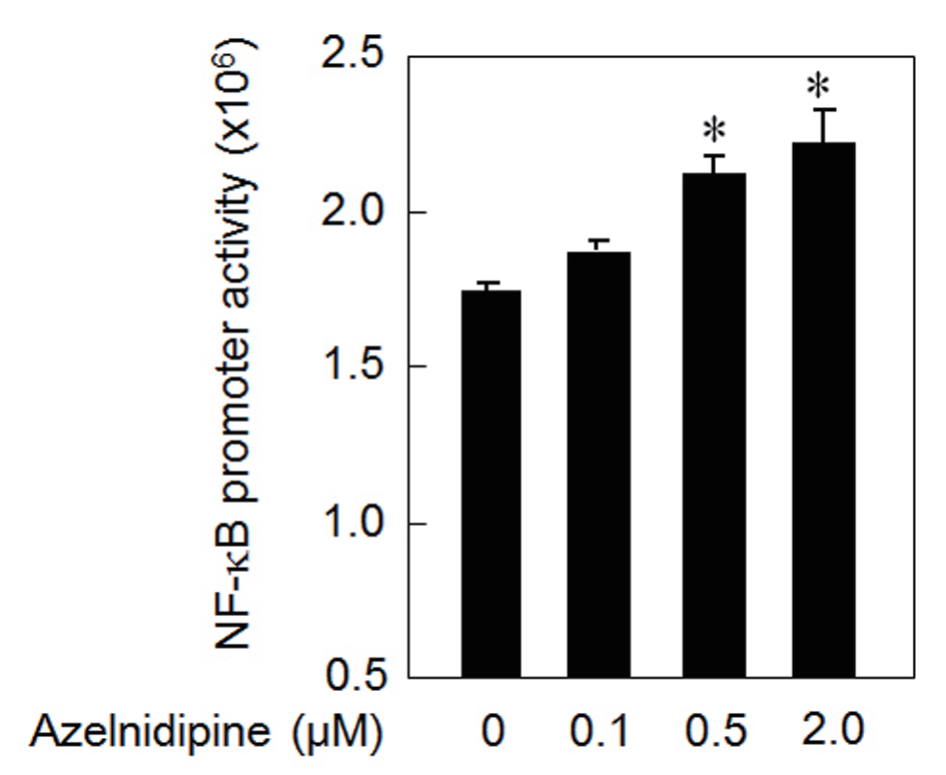
NF-κB activation using azelnidipine. *P < 0.05 vs. 0 μM azelnidipine.

In addition, to analyze the mechanism of the azelnidipine-induced secretion of VEGF, we analyzed whether azelnidipine activated NF-κB in HCSMCs. Azelnidipine induced a significant activation of NF-κB in a dose-dependent manner (Fig. 3).

### HCEC tube formation using a cell culture medium derived from CCB-treated or untreated HCSMCs

**Figure 4:**
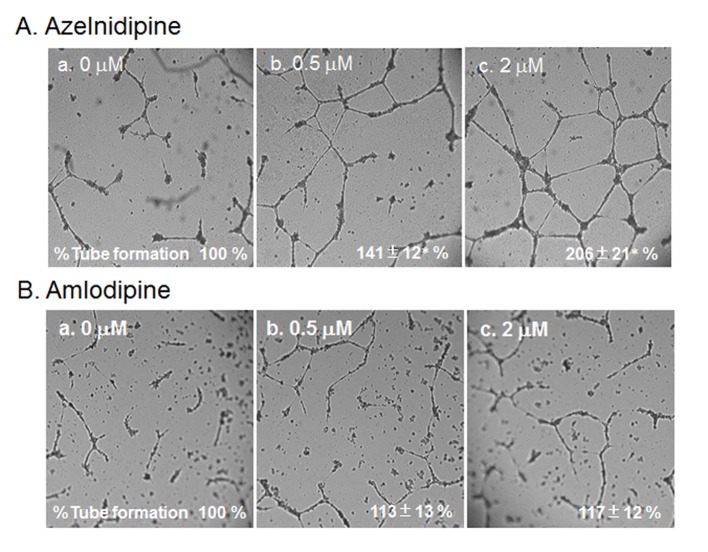
HCEC tube formation with or without the indicated concentrations of azelnidipine (A) and amlodipine (B). Representative pictures of HCECs plated on Matrigel are shown. Data show the % tube formation compared with that under no treatment (100%). *P < 0.05 vs. no treatment.

Next, we analyzed the ability of CCBs to stimulate and stabilize tube formation, with ECs cultured on Matrigel. As shown in Fig. 4, we examined whether a cell culture medium derived from CCB-treated or untreated HCSMCs could inhibit HCEC tube formation. The medium derived from azelnidipine-treated HCSMCs dose-dependently led to the formation of a capillary-like structure on the Matrigel surface, whereas amlodipine-treated HCSMCs did not promote the formation of a capillary-like structure. When 2 µmol/L of azelnidipine was incubated with HCECs on a Matrigel surface for 18 h, tube formation was not observed.

**Figure 5:**
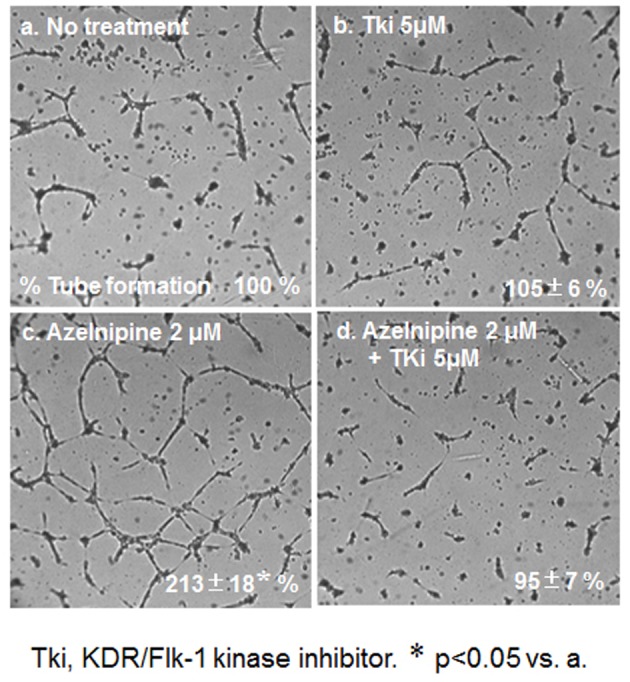
HCEC tube formation with or without 2 μM azelnidipine or 5 μM Tki. Representative pictures of HCECs plated on Matrigel are shown. Data show the % tube formation compared with that under no treatment (100%). *P < 0.05 vs. no treatment.

To analyze the mechanism of this finding, we analyzed whether azelnidipine-induced tube formation in HCECs was blocked by Tki (Fig. 5). Tube formation induced by 2 µmol/L amlodipine was blocked by Tki.

## Discussion

**Figure 6:**
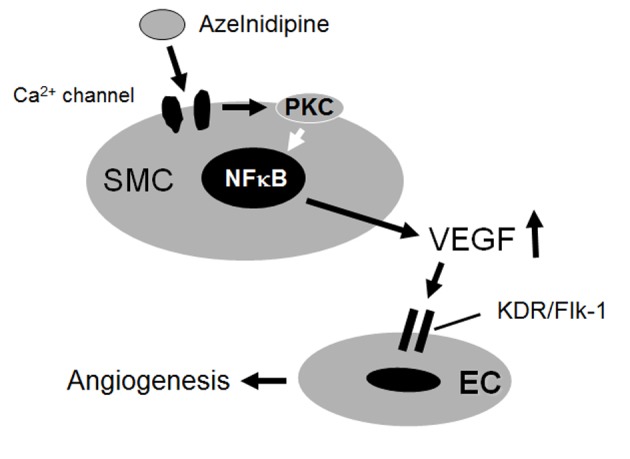
Proposed mechanisms of the effects of azelnidipine on endothelial tube formation.

In the present study, in an *in vitro* model of HCEC tube formation on a matrix gel, we showed that an increase in VEGF production from azelnidipine-treated, but not amlodipine-treated, HCSMCs through PKC may be a potent signal for inducing tube formation through the KDR/Flk-1 pathway in HCECs (Fig. 6). Azelnidipine, but not amlodipine, promoted HCEC tube formation as a molecular effect.

We found that azelnidipine promoted EC tube formation as a molecular effect. Even though the hypotensive effects of amlodipine and azelnidipine were similar throughout a 24-h administration period [14], some basic and clinical reports have suggested that the molecular effects of azelnidipine, independent of BP-lowering, are different from those of amlodipine. For example, azelnidipine, but not amlodipine, appeared to decrease intraglomerular pressure by suppressing sympathetic nerve activity in Dahl salt-sensitive rats [15]. Treatment with azelnidipine, but not amlodipine, decreased circulating advanced glycation end-products and their receptor in a BP-lowering-independent manner [16]. The number of circulating hematopoietic progenitor cells was significantly increased after the administration of azelnidipine in non-diabetic patients with essential hypertension [17]. In addition, mortality with azelnidipine was significantly lower than that with amlodipine immediately after ischemia/reperfusion in dogs [18]. Furthermore, the decreases in the left ventricular mass index with olmesartan/azelnidipine therapy were significantly greater than those with olmesartan/amlodipine therapy in hypertensive patients [19]. The increases in hematopoietic progenitor cells, a beneficial effect after ischemia/reperfusion, and the decreases in left ventricular mass by azelnidipine might be related to azelnidipine-induced endothelial protection, including the promotion of EC tube formation.

We found that a PKC inhibitor blocked the azelnidipine-induced secretion of VEGF in HCSMCs, which suggests that PKC plays a role in VEGF secretion. Our results are consistent with those of previous studies which have shown that PKC plays a role in VEGF production in response to stretching and cytokines in other biological systems [13, 20]. In addition, azelnidipine induced NF-κB activation in HCSMCs, and this activation may be involved in the azelnidipine-induced secretion of VEGF. Tong et al indicated that VEGF is upregulated by hypoxia-induced mitogenic factor (HIMF) via the PI-3Kinase/Akt-NF-κB signaling pathway in mouse lung epithelial cells [21]. In addition, HIMF induces the release of intracellular Ca^2+^ in human pulmonary artery SMCs [22]. Azelnidipine decreases intracellular Ca^2+^, and then compensatory upregulated HIMF might induce the secretion of VEGF.

Although the azelnidipine-induced secretion of VEGF promoted HCEC tube formation at up to 2 µM, a higher concentration of azelnidipine had different effects. Although 5 µM azelnidipine increased VEGF secretion from HCSMCs, this 5 µM azelnidipine-induced increase in VEGF secretion did not induce HCEC tube formation. Since 5 µM azelnidipine blocked the proliferation of HCSMCs, tube formation caused by the 5 µM azelnidipine-induced increase in VEGF secretion may be blocked by the anti-proliferative effect of 5 µM azelnidipine on HCECs. On the other hand, 5 µM amlodipine did not increase VEGF secretion and did not induce an anti-proliferative effect in HCSMCs. The effects of higher or lower concentrations of CCBs may be quite different. More detailed investigations will be required to determine the details of CCB-induced effects.

In conclusion, azelnidipine, but not amlodipine, stimulated the secretion of VEGF from HCSMCs and induced HCEC tube formation. This secretion is mediated at least in part via the activation of NF-κB. Azelnidipine may have a novel beneficial effect in improving coronary microvascular blood flow in addition to its main effect of lowering blood pressure.

## References

[1] Opie LH (1992). Should calcium antagonists be used after myocardial infarction? Ischemia selectivity versus vascular selectivity. Cardiovasc Drugs Ther.

[2] Shimamoto K, Ando K, Fujita T, Hasebe N, Higaki J, Horiuchi M, Imai Y (2014). The Japanese Society of Hypertension Guidelines for the Management of Hypertension (JSH 2014). Hypertens Res.

[3] Miura S, Karnik SS, Saku K (2011). Review: angiotensin II type 1 receptor blockers: class effects versus molecular effects. J Renin Angiotensin Aldosterone Syst.

[4] Cheng XW, Okumura K, Kuzuya M, Jin Z, Nagata K, Obata K, Inoue A (2009). Mechanism of diastolic stiffening of the failing myocardium and its prevention by angiotensin receptor and calcium channel blockers. J Cardiovasc Pharmacol.

[5] Jinno T, Iwai M, Li Z, Li JM, Liu HW, Cui TX, Rakugi H (2004). Calcium channel blocker azelnidipine enhances vascular protective effects of AT1 receptor blocker olmesartan. Hypertension.

[6] Fukuo K, Yang J, Yasuda O, Mogi M, Suhara T, Sato N, Suzuki T (2002). Nifedipine indirectly upregulates superoxide dismutase expression in endothelial cells via vascular smooth muscle cell-dependent pathways. Circulation.

[7] Brovkovych VV, Kalinowski L, Muller-Peddinghaus R, Malinski T (2001). Synergistic Antihypertensive Effects of Nifedipine on Endothelium : Concurrent Release of NO and Scavenging of Superoxide. Hypertension.

[8] Scheinowitz M (2004). Therapeutic myocardial angiogenesis: past, present and future. Mol Cell Biochem.

[9] Miura S, Fujino M, Matsuo Y, Tanigawa H, Saku K (2005). Nifedipine-induced vascular endothelial growth factor secretion from coronary smooth muscle cells promotes endothelial tube formation via the kinase insert domain-containing receptor/fetal liver kinase-1/NO pathway. Hypertens Res.

[10] Miura S, Fujino M, Matsuo Y, Kawamura A, Tanigawa H, Nishikawa H, Saku K (2003). High density lipoprotein-induced angiogenesis requires the activation of Ras/MAP kinase in human coronary artery endothelial cells. Arterioscler Thromb Vasc Biol.

[11] Miura S, Emoto M, Matsuo Y, Kawarabayashi T, Saku K (2004). Carcinosarcoma-induced endothelial cells tube formation through KDR/Flk-1 is blocked by TNP-470. Cancer Lett.

[12] Knox AJ, Corbett L, Stocks J, Holland E, Zhu YM, Pang L (2001). Human airway smooth muscle cells secrete vascular endothelial growth factor: up-regulation by bradykinin via a protein kinase C and prostanoid-dependent mechanism. FASEB J.

[13] Gruden G, Thomas S, Burt D, Lane S, Chusney G, Sacks S, Viberti G (1997). Mechanical stretch induces vascular permeability factor in human mesangial cells: mechanisms of signal transduction. Proc Natl Acad Sci U S A.

[14] Kuramoto K, Ichikawa S, Hirai A, Kanada S, Nakachi T, Ogihara T (2003). Azelnidipine and amlodipine: a comparison of their pharmacokinetics and effects on ambulatory blood pressure. Hypertens Res.

[15] Nagasu H, Satoh M, Fujimoto S, Tomita N, Sasaki T, Kashihara N (2012). Azelnidipine attenuates glomerular damage in Dahl salt-sensitive rats by suppressing sympathetic nerve activity. Hypertens Res.

[16] Nakamura T, Sato E, Fujiwara N, Kawagoe Y, Koide H, Ueda Y, Takeuchi M (2011). Calcium channel blocker inhibition of AGE and RAGE axis limits renal injury in nondiabetic patients with stage I or II chronic kidney disease. Clin Cardiol.

[17] Fukao K, Shimada K, Hiki M, Kiyanagi T, Hirose K, Kume A, Ohsaka H (2011). Effects of calcium channel blockers on glucose tolerance, inflammatory state, and circulating progenitor cells in non-diabetic patients with essential hypertension: a comparative study between azelnidipine and amlodipine on glucose tolerance and endothelial function--a crossover trial (AGENT). Cardiovasc Diabetol.

[18] Kano S, Ichihara K, Komatsu K, Satoh K (2011). Comparative effects of azelnidipine and amlodipine on myocardial function and mortality after ischemia/reperfusion in dogs. J Pharmacol Sci.

[19] Takami T, Saito Y (2011). Effects of Azelnidipine plus OlmesaRTAn versus amlodipine plus olmesartan on central blood pressure and left ventricular mass index: the AORTA study. Vasc Health Risk Manag.

[20] Berse B, Hunt JA, Diegel RJ, Morganelli P, Yeo K, Brown F, Fava RA (1999). Hypoxia augments cytokine (transforming growth factor-beta (TGF-beta) and IL-1)-induced vascular endothelial growth factor secretion by human synovial fibroblasts. Clin Exp Immunol.

[21] Tong Q, Zheng L, Lin L, Li B, Wang D, Huang C, Matuschak GM (2006). Participation of the PI-3K/Akt-NF-kappa B signaling pathways in hypoxia-induced mitogenic factor-stimulated Flk-1 expression in endothelial cells. Respir Res.

[22] Fan C, Su Q, Li Y, Liang L, Angelini DJ, Guggino WB, Johns RA (2009). Hypoxia-induced mitogenic factor/FIZZ1 induces intracellular calcium release through the PLC-IP(3) pathway. Am J Physiol Lung Cell Mol Physiol.

